# Dietary pattern and night work: metabolic syndrome in healthcare workers

**DOI:** 10.20945/2359-4292-2026-0047

**Published:** 2026-05-15

**Authors:** Francielle Lopes dos Reis, Maria Carlota Borba Brum, Júlio César Ferreira Bertoloto, Maria da Graça Rocha Penha, Sheila de Castro Cardoso Toniasso, Rodrigo Fernandes Soares, Elen Gineste Baccin, Ticiana da Costa Rodrigues

**Affiliations:** 1 Serviço de Enfermagem Ambulatorial, Hospital de Clínicas de Porto Alegre, Porto Alegre, RS, Brasil; 2 Departamento de Medicina Social, Universidade Federal do Rio Grande do Sul, Porto Alegre, RS, Brasil; 3 Faculdade de Medicina, Universidade Federal do Rio Grande do Sul, Porto Alegre, RS, Brasil; 4 Serviço de Medicina do Trabalho, Hospital de Clínicas de Porto Alegre, Porto Alegre, RS, Brasil; 5 Escola de Enfermagem, Universidade Federal do Rio Grande do Sul, Porto Alegre, RS, Brasil; 6 Departamento de Medicina Interna, Universidade Federal do Rio Grande do Sul; Serviço de Endocrinologia, Hospital de Clínicas de Porto Alegre, Porto Alegre, RS, Brasil

**Keywords:** Night work, dietary pattern, metabolic risk, chrononutrition; chronotype

## Abstract

**Objective:**

To assess the relationship between night work, metabolic syndrome (MS) prevalence, and dietary patterns in healthcare workers at a large hospital in southern Brazil.

**Subjects and methods:**

A cross-sectional study was conducted with 156 healthcare workers (90 day-shift and 66 night-shift) from July 2023 to March 2024. Sociodemographic and occupational, sleep, dietary patterns, meal timing, anthropometric data, blood pressure, and lab test data were collected.

**Results:**

Night-shift workers had higher blood pressure, lower HDL cholesterol, and 135% greater likelihood of developing MS than those who worked during the day. They consumed more fats and less fiber. Chrononutrition analysis showed night workers had later last meals (p < 0.001), longer intervals between first and last meals (p < 0.001), and shorter night fasting (p < 0.001). Ultra-processed food consumption was similar across shifts. A shorter interval between the first and last meal in night workers was linked to a 7% lower risk of MS. Findings suggest an association between night work and higher MS prevalence, with hypertension, abdominal obesity, unfavorable lipid profile, and disrupted eating timing. Circadian rhythm disruption and misaligned eating patterns, particularly prolonged eating windows and reduced nocturnal fasting, may contribute to the increased metabolic risk.

**Conclusion:**

Interventions targeting diet and chrononutrition are essential. Occupational health programs should address the specific challenges of night work.

## INTRODUCTION

Night work is widely associated with various health impacts as it deregulates circadian rhythms, which affect metabolic processes, glucose homeostasis, gastrointestinal motility, and digestive processes; influence changes in eating behavior; increase the risk of metabolic and cardiovascular diseases; and deregulates sleep ^(1,2)^. Healthcare workers are exposed to these risks due to the organization of their work, which is characterized by adverse conditions and irregular and prolonged working hours ^(2)^.

Current studies link shift work with inadequate dietary patterns ^(3,4)^. Their irregularity, sleep deprivation, and a sedentary lifestyle can significantly contribute to chronic diseases and to metabolic syndrome (MS) ^(4,5)^. Jung and cols. ^(5)^ have reported that the main risk factors for MS in the general adult population include age, higher body mass index (BMI), physical inactivity, and unhealthy eating habits.

In this context, the circadian cycle plays a key role in maintaining physiological and metabolic homeostasis, particularly influenced by exposure to natural light ^(6,7)^, regulating various physiological processes such as the sleep-wake cycle, hormone secretion, energy metabolism, insulin sensitivity, gastrointestinal motility, and appetite control ^(6-8)^. Night work promotes circadian desynchronization ^(7,9)^, with evidence demonstrating that it is associated with metabolic changes, increased systemic inflammation, impaired glycemic control, and increased cardiometabolic risk, in addition to negatively impacting sleep patterns and eating behavior ^(8-10)^.

In light of this, it is crucial to consider the contribution of chrononutrition, a field that investigates not only what individuals eat but also when they eat, taking into account the synchronization between food intake and the body’s circadian rhythms ^(8)^. Consuming food at biologically inappropriate times, such as during the night or during irregular shift schedules, can disrupt peripheral clocks, particularly in metabolic organs such as the liver, leading to weight gain and increased metabolic risk even when overall diet quality is acceptable ^(9,10)^. Shift workers often adapt their eating habits to their active hours, frequently consuming energy-dense, nutrient-poor foods during night shifts while reducing intake of fruits, vegetables, and other healthful foods ^(11,12)^. Thus, the evaluation of dietary patterns including both the nutritional composition and timing of meals is essential for understanding the risks associated with night work and developing targeted preventive strategies.

As lifestyle changes remain the most effective strategy for preventing MS and aligning biological rhythms, evidence on meal timing and eating window in workers remains limited. Therefore, this study aimed to assess the association between night work, metabolic syndrome, and dietary patterns in healthcare workers at a large hospital in southern Brazil, identifying potential opportunities for prevention and health promotion in this high-risk occupational group.

## SUBJECTS AND METHODS

### Participants and ethics

This quantitative cross-sectional study analyzed a sample of 156 employees (90 and 66 of whom worked day and night shifts, respectively) from a large hospital in southern Brazil. Participants were recruited by posters, institutional emails, and the online research bulletin board of the institution from July 2023 to March 2024. For the purposes of the study, night-shift workers were considered to work between 7pm and 7am at least 3 times a week, and day-shift workers were considered to work between 7am and 6pm on average 5 times a week.

Healthcare workers included all those who worked in the institution in health promotion and care, including support activities (administrative, hygiene, nutrition, laundry, maintenance, and surveillance). Workers who had worked at the hospital for at least one year on one of the shifts were considered as eligible participants. Exclusion criteria were pregnant women, employees on fixed-term contracts and those in the process of retiring/retired. Face-to-face meetings were held at the Clinical Research Center to collect data. The project was approved by the institution Research Ethics Committee under CAAE nº 67201723.0.0000.5327.

Overall, four self-administered questionnaires were given to participants after they signed an informed consent form (sociodemographic, alcohol consumption, physical activity, and sleep). The sociodemographic questionnaire was applied to gather personal information such as age, gender, marital status, children, schooling, work shift, time working at the institution, working hours and smoking.

The sample size was calculated considering a 95% confidence level, an expected prevalence of 25% for metabolic syndrome (p = 0.25), and a margin of error of 7%, resulting in a minimum sample size of 147 participants. A 10% increase was predicted for possible losses, totaling 162 participants as the total sample size ^(13)^. Additionally, chrononutrition variables were consulted to ensure that the planned sample was also adequate for comparing shifts on these variables ^(14)^.

### Alcohol consumption

Alcohol consumption was assessed using the Alcohol Use Disorders Identification Test questionnaire. Consumption was classified according to the score, with low risk being considered a score from 0 to 7 points; regular risk, one from 8 to 15 points; and high risk, 16 points or above ^(15)^.

### Physical activity

Individuals’ physical activity was assessed using the short International Physical Activity Questionnaire, which classifies physical activity level as sedentary (those who performed no physical activity for at least 10 continuous minutes during the week), irregularly active (those who perform physical activity below active as they fail to meet the recommended frequency or duration), and active/very active (performed vigorous activity: ≥ three days/week and ≥ 20 minutes per session, moderate or walking: ≥ five days/week and ≥ 30 minutes per session, or any activity added together: ≥ five days/week and ≥ 150 minutes/week (walking + moderate + vigorous) based on information in meters/minute/week ^(16)^.

### Sleep assessment

Daily sleep preferences were assessed using the self-assessment Munich Chronotype Questionnaire. This structured questionnaire regards waking behavior, sleep, and exposure to light on work and non-work days. Sleep duration was calculated as the end of sleep minus the beginning of sleep. Participants reported how many days during the week they had a regular work routine and the times when they go to bed, are ready for bed, fall asleep, how many minutes they need to get up, and how much time they spend outdoors ^(17)^.

The following data were calculated based on this information: sleep duration and midpoint for work and rest days, and social jetlag, which is the difference between an individuals’ “biological” and “social” clocks, which defines their social commitments throughout the day.

### Evaluation of dietary patterns

The Simplified Food Frequency Questionnaire was applied in person by trained researchers ^(18,19)^, assessing the consumption scores of fat, fiber, and ultra-processed foods. The frequency of fiber consumption was assessed of fiber consumption: whole grain bread, oats, whole grains, brown rice, beans, peas, lentils, chickpeas, soybeans, oil seeds (peanuts, walnuts, chestnuts, almonds, etc.), fresh fruit or natural juice, raw or cooked leafy greens (lettuce, cabbage, etc.), legumes and vegetables, roots, and tubers (potatoes, sweet potatoes, cassava, etc.). The following foods were included to assess fat consumption: cookies and cakes, whole milk and dairy products (yogurt, cheese, ricotta), olive oil, margarine or vegetable oils (soybean, canola, sunflower), butter or lard, fried snacks, salad with mayonnaise (potato, pasta, *salpicão*), fried foods (potatoes, cassava, polenta, etc.), industrialized snacks (salty snacks), chocolate, ice cream (fatty sweets), industrialized sweets (candies, chewing gum, etc.), eggs, and processed meats (mortadella, ham, salami, sausage, etc.).

The frequency of fat and fiber consumption was evaluated and a weight was established for each frequency. Then, the total values were added up and a score was determined for both fat and fiber consumption ^(20)^. Fiber consumption frequency had the following weights: 1× a day or more = 4, from 4 to 6× a week = 3, from 2 to 3× a week = 2, 1× a week = 1, and less than 1× a week = 0; whereas that for the frequency of fats, the following weights: 5 or more times a week = 4, 3 to 4× a week = 3, 1 to 2× a week = 2, 2 to 3× a month = 1, and less than 1× a month = 0.

Fat scores above 22 were considered as high, very high, or relatively high, whereas those below 21, as low or minimal frequency of consumption. Fiber consumption scores equal to or higher than 20 indicated a regular or adequate frequency, whereas those below 19, a low frequency of consumption ^(18,19)^.

The following ultra-processed foods were chosen for evaluation according to the classification of the food guide for the Brazilian population and the NOVA classification: margarine, cookies, cakes, industrialized salty snacks, sausages, chocolate, ice cream, industrialized sweets, soft drinks or artificial juices, fast foods, industrialized diet foods, instant noodles, ready-made food, ready-made seasonings, and mayonnaise. Daily consumption scores followed this weight: More than 3×/day = 3; 2 to 3×/day = 2; 1×/day = 1; 5 to 6×/week = 0.79; 2 to 4×/week = 0.43; 1×/week = 0.14; 1 to 3×/month = 0.07; consumes none = 0. In the analysis, a higher score indicates more frequent consumption of ultra-processed foods ^(20,21)^. Meal timing variables included the timing of the first and last meals, eating window duration, and nocturnal fasting on working days and days off.

### Anthropometric and clinical assessment

Weight and height were measured using a R/1W-200 Welmy anthropometric scale with a capacity of 200 kg and a precision of 0.100 and a Tonelli stadiometer, with a precision of 1 mm. Both measurements were used to calculate body mass index (BMI, kg/m^2)^. The cut-off points adopted for BMI were those suggested by the WHO, i.e.: normal weight (18.5-24.99); overweight (25-29.99), and obese (≥ 30.00) ^(22)^.

Blood pressure was measured from participants’ left arm as they sat after 10 minutes of rest by a Vital Signs Monitor series 300 Welch Ally digital device in the morning.

The MS outcome variable adopted diagnostic confirmation according to the criteria of the First Brazilian Guideline for the Diagnosis and Treatment of Metabolic Syndrome and the National Cholesterol Education Program Adult Treatment Panel III. Blood samples were taken for tests on free days under a 12-hour fast: fasting glycemia, glycosylated hemoglobin, total cholesterol, HDL, ultrasensitive C-reactive protein, and triglycerides at the time of anthropometric data assessment and application of the Simplified Food Frequency Questionnaire.

### Statistical analysis

The normality of continuous variables was analyzed using the Kolmogorov-Smirnov test. Continuous data were shown as means and standard deviations or medians and interquartile ranges, whereas categorical ones as absolute and relative frequencies. The Student’s *t*-test for independent samples was used to compare means between dayand night-shift workers. Homogeneity of variance was assessed using Levene’s test. In case of asymmetry, the Mann-Whitney non-parametric U test was applied. The chi-squared or Fisher’s exact tests were used to compare the proportions between worker groups. Spearman’s correlation coefficient was used to assess the association between sleep midpoint and the variables related to the interval between the first and last meal on working and off days. Poisson (categorical) or linear (numerical) regression models were applied to control for confounding factors. In case of asymmetry, the tweedie model with logarithmic transformation was applied. The data were analyzed using SPSS, version 27.0. Statistical significance was set at p ≤ 0.05.

## RESULTS

Night-shift workers had a significantly higher median age and length of service on the shift than day-shift workers, with a higher prevalence of females in both shifts. Alcohol consumption and physical activity did not show significant differences, but night-shift workers had a higher proportion of smokers (**Table 1**).

**Table 1:** Sociodemographic, behavioral, and occupational characteristics of healthcare workers at a large hospital in southern Brazil, by work shift (n = 156)

Variables	Total (156)	Daytime (90)	Night (66)	*p*
Age (years)	47 ^(42-56)^	45 ^(40-55)^	51 ^(46-56)^	**< 0.001**
Children Yes	108 (69.2%)	56 (62.2%)	52 (78.8%)	**0.041**
Sex Female Male	115 (73.7%) 41 (26.3%)	74 (82.2%) 16 (17.8%)	41 (62.1%) 25 (37.9%)	**0.006**
Marital status With partner Without partner	89 (57%) 67 (43%)	46 (51.1%) 44 (48.9%)	43 (65.2%) 23 (34.8%)	0.187
Schooling 1st/2nd grade Higher education Graduate	43 (27.6%) 44 (28.2%) 69 (44.2%)	20 (22.2%) 25 (27.8%) 45 (50%)	23 (33.3%) 19 (28.8%) 24 (36.4%)	0.158
Function Care Administrative Other	85 (54.5%) 35 (22.4%) 36 (23.1%)	42 (46.6%) 30 (33.3%) 18 (20%)	43 (65.2%) 5 (7.6%) 18 (25.2%)	**<0.001**
Length of time working 1-15 years > 15 years	91 (58.3%) 65 (41.7%)	68 (75.6%) 22 (24.4%)	23 (34.8%) 43 (65.2%)	**<0.001**
International Physical Activity Questionnaire Sedentary Active/Very active Irregularly active	23 (14.8%) 71 (45.5%) 62 (39.7%)	12 (13.3%) 41 (45.6%) 37 (41.1%)	11 (16.7%) 30 (45.4%) 25 (37.9%)	0.541
Smoking Yes	17 (10.9%)	5 (5.6%)	12 (18.2%)	**0.018**
Alcohol Use Disorders Identification Test Low risk Risk High risk	135 (86.5%) 18 (11.6%) 3 (1.9%)	81 (90%) 8 (8.9%) 1 (1.1%)	54 (​​81.8%) 10 (15.2%) 2 (3%)	0.281

Asymmetric variables are represented by medians and interquartile ranges that were assessed using the Mann-Whitney non-parametric U test. The chi-squared or Fisher’s exact tests were used for categorical variables. A significance level of p < 0.05 was adopted for all tests.

Regarding clinical assessment, night-shift workers had higher mean/median scores for various parameters such as weight, abdominal circumference; waist-to-hip ratio (WHR), systolic blood pressure and diastolic blood pressure (**Table 2**) when compared to day-shift participants.

**Table 2:** Clinical and laboratory parameters of healthcare workers at a large hospital in southern Brazil, according to work shift (n = 156)

Variables	Total (156)	Daytime (90)	Night (66)	*p*
Weight (kg)	74.60 (65.60-87.85)	70 (64.20-85.55)	80.25 (67.35-89.90)	**0.037**
BMI	27.99 (24.59-30.92)	26.55 (24.09-30.49)	28.26 (25.46-31.20)	0.159
AC	93.25 (84.0-102.5)	91 (82-100)	97.75 (89-103)	**0.010**
Female	91 (82-98)	88.8 (81-98)	93 (85.5-99.00)	0.221
Male	101 (93.3-108.5)	93.3 (88.8-111.5)	102 (97.8-106.5)	0.207
WHR	0.84 (±0.09)	0.82 (±0.09)	0.86 (±0.08)	**0.003**
Female	0.81 (±0.08)	0.80 (±0.08)	0.83 (±0.07)	0.072
Male	0.91 (±0.06)	0.91 (±0.06)	0.92 (±0.07)	0.688
Systolic BP	121 (110-134)	116 (109-126)	129.5 (117-140)	**<0.001**
Diastolic BP	75 (67-83)	72.5 (66-81)	78 (68-88)	**0.023**
CRP US	1.35 (0.60-2.80)	1.55 (0.50-3.5)	1.25 (0.60-2.60)	0.383
Total cholesterol	192 (169-222.5)	196 (166-225)	191 (169-211)	0.632
Glycated hemoglobin	5.3 (5.1-5.6)	5.2 (5.1-5.6)	5.4 (5.1-5.7)	0.067
Pre-diabetes	yes 10 (6.4%)	3 (3.3%)	7 (10.6%)	0.097
Diabetes	yes 6 (3.8%)	2 (2.2%)	4 (6.1%)	0.243

AC: abdominal circumference; HDL: high-density lipoprotein; BMI: body mass index; BP: blood pressure; CRP-US: ultrasensitive C-reactive protein; WHR: waist-to-hip ratio.

Variables with a normal distribution are shown as means and standard deviations. The Student’s *t*-test was used to analyze them. Asymmetrical variables are shown as medians and interquartile ranges and were assessed using the Mann-Whitney non-parametric U test. The chi-squared or Fisher’s exact tests were used for categorical variables. A significance level of p < 0.05 was adopted for all tests. RQC calculated mean because it is a symmetrical distribution - Levene’s Test for Equality of Variances, other variables are median, asymmetrical distribution - Independent samples Mann-Whitney U test. Pre-diabetes - Fisher’s Exact Test.

The results relating to metabolic syndrome and its components showed that hypertension occurred in 35.9% of workers, 56.1% among night-shift workers. Night-shift workers were 3.48 times more likely to have a change in blood pressure than dayshift workers, when the multivariate model was adjusted for age, gender, and time at work (PR = 3.48; 95% CI: 1.66-7.30) (**Table 3**).

**Table 3:** Diagnosis of metabolic syndrome and its components among healthcare workers at a large hospital in southern Brazil, according to work shift (N = 156)

Variables	Total n (%)	Daytime (n=90) n (%)	Night (n=66) n (%)	*p*	p^adjusted^*^^	PR (IC 95%)
Hypertension	56 (35.9)	19 (21.1)	37 (56.1)	**< 0.001**	**< 0.001**	**3.48 (1.66-7.30)**
Altered triglycerides	39 (25.0)	17 (18.9)	22 (33.3)	0.061	0.342	1.44 (0.68-3.03)
Altered HDL cholesterol	64 (41.0)	29 (32.2)	35 (53.0)	**0.014**	**0.039**	**2.20 (1.04-4.66)**
Changed AC	82 (52.6)	44 (48.9)	38 (57.6)	0.362	0.636	1.19 (0.57-2.49)
Altered blood glucose	17 (10.9)	5 (5.6)	12 (18.2)	**0.025**	0.198	2.36 (0.64-8.66)
Metabolic syndrome	41 (26.3)	15 (16.7)	26 (39.4)	**0.003**	**0.034**	**2.35 (1.07-5.15)**

* Adjusted for age, gender, and time at work.

Variables with a normal distribution are shown as means and standard deviations. The Student’s *t*-test was used to analyze them. Asymmetrical variables are shown as medians and interquartile ranges. They were assessed using the Mann-Whitney non-parametric U test. The chi-squared or Fisher’s exact tests were used for categorical variables. A significance level of p < 0.05 was adopted for all tests. To control for confounding factors, Poisson regression models (categorical) were applied. In case of asymmetry, the tweedie model with logarithmic transformation was applied.

AC: abdominal circumference; HDL: high-density lipoprotein; PR: prevalence ratio.

Overall, 41% of the sample had altered HDL cholesterol levels, with 53% of these being night-shift workers. After adjustment, they had 2.20 times lower HDL levels than day-shift workers when adjusted for age, gender, and time at work (PR = 2.20; 95% CI: 1.04-4.66). The result of the regression analysis indicated that every 1 mg/dL increase in HDL levels correlated to a 6% reduction in the probability of MS occurring in night-shift workers. Furthermore, elevated blood glucose levels were observed in 10.9% of workers, with a higher prevalence among night-shift workers. After adjustment, this difference disappeared. MS occurred in 26.3% of the total sample, 39.4% of whom were night-shift workers, who were 135% more likely to develop MS than day workers (PR = 2.35; 95% CI: 1.07-5.15) **(Table 3)**.

After adjustment by the multivariate Poisson Regression model to assess factors independently associated with MS by work shift, men had a significantly higher prevalence of MS, regardless of work shift. During the day, such probability was around 7.76 times higher in men and 2.82 times higher at night.

Sleep duration and social jetlag were similar between the groups, with no statistically significant differences. The midpoint between the start and end of sleep on working days showed a significant difference between nightand day-shift workers, even when adjusted for possible confounding factors (age, gender, children, and time at work) (**Table 4**), whereas such difference remained insignificant on rest days.

**Table 4:** Description of sleep variables by work shift in healthcare workers at a large university hospital in southern Brazil (n = 156)

Variables	Daytime (90)	Night (66)	*p*	p^adjusted^*^^
Average point of sleep on working days	2:34 (2:01-3:17)	4:48 (3:29 -7:00)	**<0.001**	**<0.001**
Average point of sleep on days off	4:01 (3:27-4:59)	4:25 (3:29-5:45)	0.084	0.098
Sleep duration on working days	6:57 (6:00-7:55)	7:29 (4:55-9:20)	0.184	0.102
Sleep duration on days off	8:00 (7:14-9:00)	8:10 (6:57-9:30)	0.541	0.392
Social jetlag	1:30 (0:44-2:01)	00:44 (00:06-2:37)	0.065	0.220

* Adjusted for age, gender, children, and time at work.

Asymmetrical variables are shown as medians and interquartile ranges. They were assessed using the Mann-Whitney non-parametric U test. The chi-squared or Fisher’s exact tests were used for categorical variables. A significance level of p<0.05 was adopted for all tests.

Night workers had greater exposure to natural light on workdays (120 min [60-240] vs. 60 min [30-180]; p < 0.022). On days off, there was no difference between the groups. In adjusted logistic regression models, exposure to natural light was not associated with the presence of metabolic syndrome (OR = 1.01; p < 0.87), low HDL (OR = 1.03; p < 0.62), or high triglycerides (OR = 1.00; p < 0.94). Work shift (night and day) was not independently associated with any of the outcomes after adjustment for other factors.

Chronotype was categorized into three groups based on MSFsc: morning (MSFsc ≤ 3:59 a.m.), intermediate (4:00-4:59 a.m.), and evening (MSFsc ≥ 5:00 a.m.) ^(23)^, considering only participants who did not depend on an alarm clock on their days off. In this sample, 56 participants were classified as morning types, 13 as intermediate types, and 37 as evening types. When stratified by work shift, daytime/full-time workers had a higher proportion of morning chronotypes (n = 41), while among night workers there was a more balanced distribution between morning (n = 15) and evening (n = 16) chronotypes, suggesting a tendency toward later sleep phases among those who work night shifts.

Regarding fat consumption, a higher frequency occurred in night-shift workers (62.1%) than in day-shift ones (51.1%). As for fiber consumption, night-shift workers had a higher frequency of low consumption (40.9%) than day-shift ones (26.7%). Despite the evident percentage variations, no statistically significant differences occurred between shifts regarding fat (p < 0.0228; adjusted < 0.242) and fiber frequency intake (p < 0.089; adjusted < 0.147).

Regarding ultra-processed foods, a similar pattern occurred in the frequency of consumption of these foods as both shifts had an estimated consumption closer to 1-2 times a week 0.28 (0.21-0.38). Small differences occurred in the consumption of sausages, with night-shift workers consuming them more frequently - from two to four times a week 0.43 (0.07-0.79), when compared to day workers who consumed them less than once a week 0.14 (0.07-0.43).

Significant differences occurred between groups for the frequency of consumption of industrialized sweets. The regression coefficient (b=-0.65; 95% CI: -1.24 to -0.05) reinforces this difference, indicating an average reduction of 0.65 points in the consumption score for night-shift workers. Both shifts recorded low values - 0.07 (0-0.43) -, but the greater variability between daytime 0.07 (0-0.43) and nighttime workers - 0.07 (0-0.14) - highlights a pattern of reduced consumption frequency in this work shift. The consumption of soft drinks and artificial juices showed a difference close to the limit of significance (p < 0.052). Statistical analysis showed no significant differences in most of the food variables when adjusted for age, gender, and time at work.

The first and last meal on working days between shifts and non-working days statically differed (**Table 5**), suggesting that night-shift workers have a longer eating window on work and off days than day-shift workers. The regression analysis found that reducing the interval between the first and last meal for night-shift workers is associated with a 7% reduction in their risk of MS.

**Table 5:** Comparison of dietary pattern and chrononutrition variables between night and day shifts (n = 156)

Variables	total sample n (%)	Daytime (n=90) n (%)	Night (n=66) n (%)	*p*	p^adjusted^*^^
Fat^**^ Low/Minimum High/Very High/Relatively High	69 (44.2%) 87 (55.8%)	44 (48.9%) 46 (51.1%)	25 (37.9%) 41 (62.1%)	0.228	0.242
Fiber^**^ Adequate Consumption Low consumption	105 (67.3%) 51 (32.7%)	66 (73.3%) 24 (26.7%)	39 (59.1%) 27 (40.9%)	0.089	0.147
Median consumption^***^ of ultra-processed foods	0.28 (0.22-0.38)	0.28 (0.21-0.38)	0.28 (0.21-0.38)	0.971	0.749
Difference in time between first and last meal (hours) on working days	13:52 (12:00-16:00)	13:00 (12:00-14:30)	16:00 (13:00-18:00)	**<0.001**	**<0.001**
Difference in time between first and last meal (hours) on days off work	12:00 (11:00-13:00)	12:00 (11:00-13:00)	12:30 (11:00-14:00)	**0.045**	**0.030**
Night fasting on working days	10:00 (8:00-11:30)	11:00 (9:12-12:00)	8:00 (6:00-10:00)	**<0.001**	**<0.001**
Night fasting on days off	12:00 (11:00-13:00)	12:00 (11:00-13:00)	12:00 (10:00-13:00)	0.401	0.992
Number of meals in the working day	4 ^(4-5)^	4 ^(4-5)^	4 ^(4-5)^	0.489	0.546
Number of meals on days off	4 ^(4-5)^	4 ^(3-5)^	4 ^(4-5)^	0.165	0.149

* Adjusted for age, gender, children, and time at work.

** Fat score greater than 22 - high, very high, relatively high, less than 21 low/minimal consumption; Fiber - equal to or greater than 20 - regular/adequate, less than 19 - low consumption.

*** Daily consumption score of ultra-processed foods: More than 3×/day = 3; 2 to 3×/day = 2; 1×/day = 1; 5 to 6×/week = 0.79; 2 to 4×/week = 0.43; 1×/week = 0.14; 1 to 3×/month = 0.07; consumes none = 0.

Regarding nocturnal fasting on working days, night-shift workers had a shorter fasting time than day-shift ones [8:00 (6:00-10:00) vs. 11:00 (9:12-12:00) p < 0.001], after adjusting for possible confounding factors (age, gender, and time at work). On days off, both groups had a similar duration of overnight fasting [12:00 (10:00-13:00) vs. 12:00 (11:00-13:00) p < 0.401] (**Table 5**). Regarding the number of meals, no differences occurred between the shifts regarding the number of meals on work or off days (**Table 5**).

When analyzing the correlation between sleep midpoint and variables related to the difference between the first and last meal on work and off days, no significant correlation occurred for either daytime or nighttime individuals. However, on days off, a negative and significant correlation occurred between the difference in meal times and the midpoint of sleep for nocturnal individuals (rs = -0.250; p < 0.043), suggesting a possible relation between the meal interval and the midpoint of sleep on days off. As for night-time fasting, no significant correlations occurred for daytime (rs = 0.083; p < 0.437) or nighttime individuals (rs = 0.148; p < 0.235) on working days, indicating that night-time fasting has no statistically relevant association with sleep midpoint in either group.

## DISCUSSION

Our results suggest that night work is significantly associated with an increase in the frequency of metabolic syndrome and its individual components (hypertension, increased body measurements such as weight, abdominal circumference and waist-to-hip ratio), which indicate greater accumulation of body fat, similarly to other studies ^(24)^. Moreover, an observational study of retired workers suggested that the adverse effects of night shifts on cardiometabolic function may persist after retirement, with differences between men and women ^(25)^. Another cross-sectional study highlighted that specific characteristics of night-shift work, such as the frequency and duration of shifts, are associated with high metabolic risk factors, such as increased BMI and waist circumference ^(25,26)^.

Research has found significant associations between higher night-shift workload and higher blood pressure levels than lower cumulative night-shift workload ^(26,27)^. A systematic review and meta-analysis offers sufficient evidence for a potential link between permanent night-shift work and an increase in blood pressure values, reinforcing the data in ^(28)^. The reversal of working hours may be associated with disruption of the circadian cycle and chronic stress. Sleep deprivation and disturbances in rest schedules may configure key factors explaining these changes ^(29-35)^.

Social jet lag was greater for daytime workers, but this can be justified by the greater variation between sleep schedules on workdays and days off, as night workers tend to maintain later schedules consistently. These data are consistent with previous studies showing that circadian misalignment is dependent on both chronotype and work shift demands ^(36)^.

Despite the circadian difference between shifts, social jet lag and midpoint of sleep were not associated with the metabolic outcomes assessed. Greater exposure to natural light among night-shift workers may also mitigate some of the circadian misalignment, as sunlight is a powerful synchronizer of biological rhythm and improves adaptation to night shifts. This compensatory effect was described ^(37)^, and confirmed in working populations ^(36)^, who demonstrated that exposure to natural light after the night shift helps realign the circadian rhythm, increasing alertness and reducing the negative effects of dysregulation. Even though night workers have greater exposure to natural light, this did not impact the diagnosis of MS or the lipid profile. Studies show the greater impact of artificial light on HDL and triglyceride levels ^(38,39)^.

Night workers have a 12-hour night shift (7:00 p.m. to 7:00 a.m.) with a 60-hour break, which can directly influence both the time of exposure to natural light and the interpretation of circadian patterns. This pattern implies that exposure to natural light on workdays occurs in the morning and during the commute at the end of the night shift, when solar irradiance is already high. In contrast, the rest window (60 hours) means that, on subsequent days, exposure to natural light is similar to that observed among day workers, which may explain the absence of differences between the groups on days off.

Studies suggest that engaging in physical activity at times misaligned with the circadian rhythm may be associated with sleep disturbances and circadian misalignment among shift workers ^(40)^. Although it was not possible to assess the timing of physical activity in the present sample, since only the IPAQ was used to measure activity level, it is important to recognize that both the amount and timing of physical activity can influence the metabolic and circadian health of this population.

The later night-shift workers sleep, the shorter the interval between their first and last meal on off days, suggesting that the way they organize their meals may influence when they go to sleep, showing a more regular behavior on days off or a response considered more natural to the biological rhythm outside the pressure of working hours.

Our analysis of eating patterns suggests that these workers often consume more fat and less fiber than daytime workers, suffer a greater impact on the time between the first and last meal and on nighttime fasting, and show disorganized schedules and less fasting on working days. Changes in meal times can negatively impact glucose tolerance and contribute to insulin resistance since eating at night is associated with glycemic peaks and an increased risk of diabetes. This finding suggests that meal timing may represent a modifiable target for metabolic risk reduction among night-shift healthcare workers. Moreover, the higher frequency of fat consumption and lower fiber intake in these workers reinforces the negative effects on glucose metabolism, which can contribute to the development of cardiometabolic dysfunctions. The prevalence of hypertension in these workers may also increase by inadequate dietary patterns, such as the high consumption of sodium in ultra-processed foods ^(41-43)^.

A study has explored fasting as an intervention to reduce the impact of simulated night shifts on glucose metabolism. Results showed that overnight fasting can improve insulin sensitivity and glucose tolerance when compared to meals or snacks during the night ^(44)^. Moreover, daytime eating at appropriate times can prevent internal circadian dysregulation ^(45)^.

In this specific sample, it was impossible to correlate the composition and quantity of the diet with clinical and laboratory variables, but eating times seem to constitute a way of predisposing these workers to a worse quality dietary pattern considering the circadian cycle ^(14,46)^. Thus, an increased calorie balance during the 24-hour period of the day due to eating at night could predispose to the risk of obesity and metabolic disorders ^(47,48)^.

The influence of environmental factors, such as lack of places to eat, limited healthy food choices, lack of time, easy access to unhealthy foods, and the influence of colleagues’ preferences contribute to inadequate food consumption in night-shift workers ^(49-53)^, leading to poor food quality and increased calorie intake ^(54-57)^.

Lack of knowledge can also contribute to poor choices, making it necessary to consider nutritional education. A well-structured dietary intervention in the workplace that combines nutritional education and environmental dietary modification can help to acquire knowledge and increase consumption of suitable foods ^(58)^.

Restricting eating time during the day can also offer an effective strategy for dietary and glycemic control and weight loss, positively affecting insulin resistance and 24-hour glucose levels in people with obesity ^(59)^.

The large prospective study Palomar-Cros et al. carried out in France concluded that a late first meal is associated with a higher incidence of type 2 diabetes mellitus. They state that a circadian eating behavior with an early first meal (before 8 a.m.) and an early last meal (before 7 p.m.) may be beneficial in reducing the incidence of type 2 diabetes mellitus ^(60)^. Another large study carried out in Spain to analyze eating time patterns associated with body weight found that a later first meal time was associated with a higher BMI, whereas a longer night fast was associated with a lower BMI in cross-sectional and longitudinal analyses ^(61)^.

The night-shift workers in the studied sample have a chrononutritional profile with a greater difference between meals and less fasting at night, so it is essential to consider these aspects in the prevention of metabolic diseases. The development of prevention strategies must comprehensively consider biological, behavioral, and social aspects and focus on the factors that can interfere with maintaining health.

This study has some limitations that should be considered. The cross-sectional design prevents establishing causality; data collection at a single center limits the generalizability of the findings; and sleep and dietary patterns were assessed using self-administered questionnaires, which are subject to memory and perception biases. Additionally, voluntary participation may have introduced self-selection bias, as in-person attendance may have favored workers with more flexible routines. Despite these limitations, the study provides clinically relevant insights into modifiable eating behaviors associated with metabolic risk in night-shift healthcare workers. The integration of sleep characteristics, dietary patterns, and metabolic indicators allows the identification of potential targets for workplace-based prevention and intervention strategies, particularly in occupational groups exposed to long-term cardiometabolic risks.

In conclusion, there are significant nutritional challenges for night workers due to factors that may be associated with work organization and/or personal issues. The research results point to the importance of interventions targeted at this group of workers. Implementing strategies that meet nutritional needs and promote healthy habits is essential to reduce risks and improve quality of life. In addition, the creation and/or adaptation of occupational health policies can play a key role in reducing the negative impacts of night work. Measures such as flexible meal times, providing healthier food options in the workplace, and implementing nutrition education programs can contribute to improving the dietary patterns of these professionals. Other strategies could include reducing the workload for those exposed to long shifts, ensuring adequate rest periods, and promoting regular medical follow-up, with the aim of minimizing the effects of circadian cycle reversal and reducing the prevalence of metabolic diseases.

Further studies are needed to gain a deeper understanding of the long-term associations between dietary patterns, daily food consumption time, and their effects on the health of these workers, including other public and private institutions. In particular, it is essential to investigate the role of this dietary pattern in the development of metabolic diseases, such as obesity. Meal timing strategies may represent an additional tool for metabolic risk management in night-shift healthcare workers.

## Data Availability

data from the ELSA-Brasil Study is subject to restricted access.
