# Erratum: Dietary pattern and night work: metabolic syndrome in healthcare workers

**DOI:** 10.20945/2359-4292-2026-0072

**Published:** 2026-07-03

In the article **“Dietary pattern and night work: metabolic syndrome in healthcare workers”**, published in Archives of Endocrinology and Metabolism, DOI: 10.20945/2359-4292-2026-0047:

Page 1, Authors

Where it reads:

Francielle Lopes dos Reis^1*^

https://orcid.org/0000-0003-2134-1157

Maria Carlota Borba Brum^2^

https://orcid.org/0000-0001-8124-2527

Júlio César Ferreira Bertoloto^3^

https://orcid.org/0009-0009-5597-5300

Maria da Graça Rocha Penha^4^

https://orcid.org/0009-0008-5723-6408

Sheila de Castro Cardoso Toniasso^4^

https://orcid.org/0000-0001-8692-7330

Rodrigo Fernandes Soares^5^

https://orcid.org/0009-0004-3887-2645

Elen Gineste Baccin^1^

https://orcid.org/0000-0001-6669-6686

Ticiana da Costa Rodrigues^6^

https://orcid.org/0000-0001-9254-3712

^1^ Serviço de Enfermagem Ambulatorial, Hospital de Clínicas de Porto Alegre, Porto Alegre, RS, Brasil

^2^ Departamento de Medicina Social, Universidade Federal do Rio Grande do Sul, Porto Alegre, RS, Brasil

^3^ Faculdade de Medicina, Universidade Federal do Rio Grande do Sul, Porto Alegre, RS, Brasil

^4^ Serviço de Medicina do Trabalho, Hospital de Clínicas de Porto Alegre, Porto Alegre, RS, Brasil

^5^ Escola de Enfermagem, Universidade Federal do Rio Grande do Sul, Porto Alegre, RS, Brasil

^6^ Departamento de Medicina Interna, Universidade Federal do Rio Grande do Sul; Serviço de Endocrinologia, Hospital de Clínicas de Porto Alegre, Porto Alegre, RS, Brasil

Read:

Francielle Lopes dos Reis^1*^

https://orcid.org/0000-0003-2134-1157

Maria Carlota Borba Brum^2^

https://orcid.org/0000-0001-8124-2527

Júlio César Ferreira Bertoloto^3^

https://orcid.org/0009-0009-5597-5300

Maria da Graça Rocha Penha^4^

https://orcid.org/0009-0008-5723-6408

Sheila de Castro Cardoso Toniasso^4^

https://orcid.org/0000-0001-8692-7330

Rodrigo Fernandes Soares^5^

https://orcid.org/0009-0004-3887-2645

Elen Gineste Baccin^1^

https://orcid.org/0000-0001-6669-6686

Ticiana da Costa Rodrigues^6^

https://orcid.org/0000-0001-9254-3712

Camila Pereira Baldin^1^

https://orcid.org/0000-0001-6134-338X

Robson Martins Pereira^1^

https://orcid.org/0009-0006-0347-3913

^1^ Serviço de Enfermagem Ambulatorial, Hospital de Clínicas de Porto Alegre, Porto Alegre, RS, Brasil

^2^ Departamento de Medicina Social, Universidade Federal do Rio Grande do Sul, Porto Alegre, RS, Brasil

^3^ Faculdade de Medicina, Universidade Federal do Rio Grande do Sul, Porto Alegre, RS, Brasil

^4^ Serviço de Medicina do Trabalho, Hospital de Clínicas de Porto Alegre, Porto Alegre, RS, Brasil

^5^ Escola de Enfermagem, Universidade Federal do Rio Grande do Sul, Porto Alegre, RS, Brasil

^6^ Departamento de Medicina Interna, Universidade Federal do Rio Grande do Sul; Serviço de Endocrinologia, Hospital de Clínicas de Porto Alegre, Porto Alegre, RS, Brasil

The authors reported that the names of Camila Pereira Baldin and Robson Martins Pereira were inadvertently omitted from the author list during the production process. This correction refers exclusively to the authorship information and does not affect the scientific content, results, analyses, or conclusions of the study.

**Editor in chief:**

César Boguszewski

https://orcid.org/0000-0001-7285-7941

